# Investigating musculoskeletal health and wellbeing; a cohort study protocol

**DOI:** 10.1186/s12891-020-03195-4

**Published:** 2020-03-21

**Authors:** Bonnie Millar, Daniel F. McWilliams, Abhishek Abhishek, Kehinde Akin-Akinyosoye, Dorothee P. Auer, Victoria Chapman, Michael Doherty, Eamonn Ferguson, John R. F. Gladman, Paul Greenhaff, Joanne Stocks, Ana M. Valdes, David A. Walsh

**Affiliations:** 1 NIHR Biomedical Research Centre, Academic Rheumatology, University of Nottingham Clinical Sciences Building, City Hospital, Nottingham, NG5 1PB UK; 2 Division of ROD, School of Medicine, University of Nottingham, Nottingham, UK; 3 Pain Centre Versus Arthritis, University of Nottingham, Nottingham, UK; 4 Division of Clinical Neuroscience, School of Medicine, University of Nottingham, Nottingham, UK; 5 School of Life Sciences, University of Nottingham, Nottingham, UK; 6 School of Psychology, University of Nottingham, Nottingham, UK; 7 Rehabilitation, Ageing and Wellbeing, School of Medicine, University of Nottingham, Nottingham, UK; 8 Division of Physiology, Pharmacology and Neuroscience, University of Nottingham, Nottingham, UK; 9 Sherwood Forest Hospitals NHS Foundation Trust, Sutton in Ashfield, Nottinghamshire, UK

**Keywords:** Pain, Osteoarthritis, Frailty, Disability, Cohort, Knee

## Abstract

**Background:**

In an ageing population, pain, frailty and disability frequently coexist across a wide range of musculoskeletal diagnoses, but their associations remain incompletely understood. The Investigating Musculoskeletal Health and Wellbeing (IMH&W) study aims to measure and characterise the development and progression of pain, frailty and disability, and to identify discrete subgroups and their associations. The survey will form a longitudinal context for nested research, permitting targeted recruitment of participants for qualitative, observational and interventional studies; helping to understand recruitment bias in clinical studies; and providing a source cohort for cohort randomised controlled trials.

**Methods:**

IMH&W will comprise a prospective cohort of 10,000 adults recruited through primary and secondary care, and through non-clinical settings. Data collection will be at baseline, and then through annual follow-ups for 4 years. Questionnaires will address demographic characteristics, pain severity (0–10 Numerical Rating Scale), pain distribution (reported on a body Manikin), pain quality (McGill Pain Questionnaire), central aspects of pain (CAP-Knee), frailty and disability (based on Fried criteria and the FRAIL questionnaire), and fracture risk. Baseline characteristics, progression and associations of frailty, pain and disability will be determined. Discrete subgroups and trajectories will be sought by latent class analysis. Recruitment bias will be explored by comparing participants in nested studies with the eligible IMH&W population.

**Discussion:**

IMH&W will elucidate associations and progression of pain, frailty and disability. It will enable identification of people at risk of poor musculoskeletal health and wellbeing outcomes who might be suitable for specific interventions, and facilitate generalisation and comparison of research outcomes between target populations. The study will benefit from a large sample size and will recruit from diverse regions across the UK. Purposive recruitment will enrich the cohort with people with MSK problems with high representation of elderly and unwell people.

**Trial registration:**

Clinicaltrials.gov NCT03696134. Date of Registration: 04 October 2018.

## Introduction

Improvements in public health and medical treatments over recent decades have contributed to increased life expectancies and increasingly ageing populations. Musculoskeletal (MSK) conditions, such as osteoarthritis and spinal pain are amenable to treatment, but often are not cured, and their prevalence therefore increases with increasing age. MSK conditions contribute importantly to pain, frailty and disability. Indeed, osteoarthritis and spinal pain are amongst the most common causes of disability in the UK and globally [1–3]. With associated comorbidities and multimorbidity (cardiovascular, metabolic, obesity, neurological, mental health), MSK conditions increasingly threaten personal independence and challenge healthcare budgets. Each year, 20 % of UK population consults primary care for MSK conditions [4]. Increasing MSK problems are associated with increased risk of frailty [5], which in turn is associated with more frequent falls, fractures and associated morbidities [6].

Pain, frailty and disability are problems that may be shared between diagnostic groups, and with people who do not have a discrete pathological diagnosis. Pain is an unpleasant sensory and emotional experience associated with actual or potential tissue damage, or described in terms of such damage [7, 8]. Frailty has been defined as a state of increased vulnerability resulting from aging-associated decline in reserve and function across multiple physiologic systems such that the ability to cope with every day or acute stressors is compromised [6, 9]. Disability has been defined in UK law as a physical or mental impairment that has a substantial and long term adverse effect on the ability to carry out normal day to day activities [10]. These constructs may only weakly be associated with underlying diagnosis. For example, guidance for physical activity [11] or for the prevention and management of frailty [12] might equally apply to clinical and non-clinical populations. Interventions might include pharmacological, physiotherapeutic, occupational, psychological, and educational approaches. Interventions aim to maintain physical and psychological wellbeing into later life, but only rarely offer permanent cure for MSK problems. Even with optimal adherence to treatment, MSK health and wellbeing frequently decline, leading to loss of independence and substantial personal and societal burden.

Limited effectiveness of existing interventions for MSK problems can be attributed in part to our incomplete understanding of the mechanisms which link MSK pain, frailty and disability. Pain results from complex interactions between MSK pathology, peripheral and central neuronal sensitisation, psychological and social factors [13, 14]. Frailty comprises both a physiological state (for example, as may be revealed by grip strength or weight loss) and an accumulation of multiple deficits which compromise homeostatic reserve. Loss of skeletal muscle mass and strength (sarcopenia), and pain each may importantly contribute to frailty. Pain, frailty, MSK pathology and psychological distress each can contribute to the disability associated with MSK problems.

Despite the overlapping mechanisms that might link pain, frailty and disability [5], each may be considered a discrete problem of importance both to individuals and to society. Pain is not always associated with frailty, and factors such as sarcopenia or osteoporosis are not necessarily direct causes of pain. Exercise might increase muscle mass and strength but also can increase joint pain in the short term. Holistic assessment can identify the problems which are of importance for an individual, and inform their optimal management. However, there remains uncertainty on how best to combine and target interventions to multiple problems in order that the individual may achieve the best possible outcome.

Randomised controlled trials (RCTs) provide the highest quality evidence for superiority of one intervention over another. However, results of RCTs often translate suboptimally into clinical care, due to lack of generalisability from study populations and research contexts into the real world. Cohort studies provide additional and complementary evidence alongside that available from RCTs. Data collection using standardised protocols may be repeated over long time periods, addressing multiple domains, and engaging large numbers of participants in diverse contexts. Cohort studies are ideally suited to explore long-term outcomes, risk factors and associations, and heterogeneity within populations. Previous cohort studies have provided evidence that MSK pain might either improve or progress [15–19], and have empirically identified subgroups of participants [20, 21] and discrete clinical trajectories [22–25] associated with better or worse outcomes. However, reversibility and outcome prediction for frailty remain largely unexplored, and previous cohorts have not investigated links between pain, disability and frailty in people with MSK problems.

Cohort studies can facilitate the design, execution and interpretation of RCTs and other clinical studies [26]. Identification and recruitment of eligible participants can be a limiting factor for the successful execution of an RCT. Cohorts, by comprising large populations of well-phenotyped participants, may facilitate identification of people who both meet eligibility criteria, and have interest in participation in research studies. RCTs of complex interventions raise difficulties with the design of appropriate control interventions, and comparisons with `usual care’ are often made [27]. However, `usual care’ within an RCT might not approximate normal clinical practice. Research participants might be provided with information about treatment options that are novel or not otherwise accessible. Allocation to what may be seen by participants as inferior `usual care’ might lead to dissatisfaction and this might be a barrier to achieving an optimal outcome. Cohorts can provide data from contemporary usual care on participants who are matched for demographic and clinical characteristics to participants recruited to active treatment. In this Cohort RCT design, cohort participants are informed that their data may be used to provide control comparison to intervention studies, without providing details of those interventions, and without raising participant expectations that they might receive additional or novel interventions [28, 29]. Recruitment of participants from within cohorts can also permit follow up beyond the duration of an RCT, permitting measurement of outcomes that might be associated with initial treatment allocation and with trial participation. Furthermore, recruitment from within cohort studies can provide insight into recruitment bias, identifying the extent to which participants recruited to nested studies might differ from source populations, and facilitating generalisation of RCT findings to the target population [30].

IMH&W aims to elucidate associations and progression of pain, frailty and disability. It will enable identification of people at risk of poor MSK health and wellbeing who might be suitable for specific interventions, and facilitate generalisation and comparison of research outcomes between target populations.

### Objectives

The primary objective is to measure and characterise the development and progression of pain, frailty and disability and their associations in a cohort of adults with MSK problems.

Secondary objectives are to:

1. Identify discrete participant subgroups and their associations, based on baseline characteristics and on pain, frailty and disability trajectories.
2. Facilitate nested qualitative, observational and interventional research studies by providing information required for study design, and by permitting the identification and recruitment of eligible research study participants.
3. Characterise the longitudinal context and recruitment biases of nested research studies in order to better generalize from their findings, to provide long-term observational follow up data, and to provide a source cohort for cohort randomized controlled trials (RCTs).

## Methods/design

### Study design and setting

IMH&W is a prospective, observational cohort study. Adults with or at risk of MSK problems will be recruited from multiple primary and secondary care and community populations in the UK. Waves of data collection will use self-report questionnaires at baseline, and annually for 4 years after recruitment. Some IMH&W participants will also be invited to participate in nested research studies.

### Ethical considerations

All aspects of this study were approved by the Central London Research Ethics Committee (REC ref. 18/LO/0870) and will be performed in accordance with the Declaration of Helsinki, the principles of Good Clinical Practice (GCP) and the UK Policy Framework for Health and Social Care Research, 2018 [31].

Participation requires ongoing informed consent, documented at baseline through a signed consent form, and at each follow up by written confirmation of willingness for continued participation and further contact. Participants will be informed about the study through a written Participant Information Sheet (PIS), supported by face:face or telephone discussion with a member of the research team where requested. Participants will consent to their data being stored for use in research and for the research team to access their medical records for research. Participants may also consent to be contacted by the research team with information about additional research studies, and for use of their data for comparison with data from other research studies, including intervention groups in cohort RCTs.

Entry into the study is voluntary and any treatment and care received at the time or in the future will not be affected by the decision whether or not to participate. Participation in this study does not require nor exclude participation in other research projects. Participants may, at any time, withdraw consent to participate in IMH&W without impact on their clinical care or eligibility to participate in other research studies, although their individual data cannot be removed from anonymised analyses undertaken prior to withdrawal of consent.

### Public and patient involvement and engagement

All participant-facing documentation, including PIS and Consent Form was prepared in partnership with members of the NIHR Nottingham BRC Musculoskeletal Theme’s Public and Patient Involvement and Engagement Advisory Group. Advisory group members identified pain, disability and frailty as important areas of concern, and provided feedback through face:face meetings, semi-structured telephone interviews and by email. Participants will be updated on research progress through newsletters and websites (https://nottinghambrc.nihr.ac.uk, blogs.nottingham.ac.uk/MSK.

and www.nottingham.ac.uk/paincentre). Research results will be disseminated through websites, Twitter feeds, public engagement events organised by the NIHR Nottingham BRC and Pain Centre Versus Arthritis and, where appropriate, local and national media. Participant feedback will help us to design future studies that optimally respect and respond to the needs of research participants.

### Participants

#### Inclusion criteria

All of the following:

- Aged 18 or over,
- Able to give informed consent.
- Have, or are at risk of developing, frailty, MSK pain or disability.

#### Exclusion criteria

Any of the following:

- Persons who do not adequately understand verbal information in written English, or who have special communication needs, will be excluded due to non-availability of validated version of the questionnaires in languages other than English or special communication formats.
- Major non-MSK conditions likely to preclude follow up or eligibility for nested research studies; receiving dialysis and/or home oxygen, diagnosis of terminal cancer, unstable angina, severe heart failure, serious mental illness, dementia end of life care pathway.

#### Rolling enriched recruitment

A rolling, enriched recruitment strategy will be adopted to facilitate nested research studies and provide a context for future cohort RCTs (for which eligibility criteria have not been defined prior to the first phase of recruitment to IMH&W). IMH&W will be used as an environment for cohort RCTs, when targeted rolling recruitment to IMH&W will invite potential participants who meet eligibility criteria for that RCT. Furthermore, participants in other research studies who have expressed a willingness for further contact about research participation will be invited to participate in IMH&W. In these ways, IMH&W may provide control data for cohort RCTs and provide longitudinal data beyond completion of shorter research studies. Baseline characteristics of the IMH&W cohort will therefore change over time according to ongoing research needs, while maintaining a focus on MSK health and wellbeing.

Diverse recruitment pathways will facilitate the identification of participants from a range of backgrounds and contexts (Table 1). Primary care will be the predominant recruitment pathway, and recruitment will also be through the community and secondary care health services. Participants may be identified through screening of primary or secondary care patient lists, might have attended clinics, and might be on waiting lists for treatment (e.g. outpatient, physiotherapy and inpatient). Information about IMH&W may be on display as posters in public (e.g. clinical, research or leisure) areas, media or online, including contact details that enable participants to make direct contact with the IMH&W research team.

**Table 1:** Schedule for recruitment and assessments

	Screening	Time points				
		Baseline	t1	t2	t3	t4
**Recruitment**						
Primary care databases	✓					
Secondary care waiting lists	✓					
Public posters	✓					
Media or online	✓					
Outpatient visit	✓					
Other research studies	✓					
**Enrolment**						
Check eligibility	✓	✓	✓	✓	✓	✓
Signed consent		✓				
**Assessments**						
Demographics and lifestyle		✓	✓	✓	✓	✓
Pain phenotyping						
*Joint pain, McGill pain questionnaire*^a^*, CAP-Knee*		✓	✓	✓	✓	✓
Frailty classification/scoring						
*FRAIL, FiND, Fried criteria*^a^		✓	✓	✓	✓	✓
Medical conditions and treatments						
*FRAX*^a^*, comorbidities, medical treatments*		✓	✓	✓	✓	✓
Wellbeing						
Mental health and activity		✓	✓	✓	✓	✓

^a^selected items. *CAP-Knee;* Central Aspects of Pain-Knee questionnaire, *FRAIL;* Fatigue, Resistance, Ambulation, Illnesses, & Loss of Weight questionnaire, *FiND;* Frail Non Disabled scale

Initial recruitment from the community will be enriched at pre-screening with people with frailty by invitations sent to people based on e-Frailty Index (eFI) scores of 0.12 or more (the threshold for mild frailty) recorded by their GP [32, 33]. eFI is commonly documented within primary care in the UK [34]. Initial recruitment will also be enriched at pre-screening to support ongoing research on osteoarthritis knee pain, based on knee pain in people aged > 50 years [19]. Recruitment will be geographically targeted to include `seldom heard groups’ that might not typically participate in clinical research, and local populations where nested studies require attendance for face:face assessment or intervention.

Pre-screening and primary contact for recruitment will be by an individual with recognised legitimate access to personal details. This may be NHS staff who form part of the participants’ normal care team, or, for participants in previous research, a member of the relevant research team. Potential participants will be given/sent information about the study, the baseline questionnaire and the consent form. Information may be provided by post, during a healthcare visit, or through an on-line link. Participants who respond but are found at screening of the submitted information to not meet eligibility criteria will be thanked for their interest, but not included within the cohort.

### Data sources and measurement

The baseline and follow up IMH&W questionnaires have been developed using previously validated instruments (Supplementary materials 1 and 2). Items were included in order to identify and measure participant demographics and clinical characteristics that are relevant to MSK pain, frailty and disability. The questionnaire was designed to be brief in order to maximise recruitment rates and minimise burden to potential participants, while providing the key information required for screening in order to recruit participants to more detailed research studies in the future. The follow up IMH&W questionnaire (Supplementary materials 2) comprises items from the baseline questionnaire, excluding redundant items (e.g sex, which would not change from baseline, and recall of pain from the previous year). Follow up questionnaires might be supplemented by additional items dependent on requirements of ongoing cohort RCTs and other studies. At each successive wave of follow up, data collection may be modified to meet the needs of current MSK health and wellbeing research.

Demographic characteristics will be recorded at baseline; age, sex, ethnicity, height and weight (current and 1 year ago), smoking status, and alcohol consumption. Morbidities and current medication use (prescribed or over the counter) will be recorded using a standardised list for most commonly reported medications plus supplementary free text. Characteristics that have been associated with central pain sensitisation will be assessed using the Central Aspects of Pain in the Knee (CAP-Knee) scale [6]. Pain distribution will be recorded using a whole body pain manikin [28], and pain quality using the word descriptors from the McGill Pain Questionnaire [28]. For those who report pain or aching in any joint over the past 4 weeks, the most bothersome joint and its pain intensity (Numerical Rating Scale from 0 (no pain) to 10 (pain as bad as could be) [27]) will be recorded. Additional aspects of frailty and disability will be measured using 3 items from the Fatigue, Resistance, Ambulation, Illnesses, & Loss of Weight (FRAIL) questionnaire addressing Fatigue, Resistance and Ambulation [3], an item from the FiND questionnaire addressing physical activity [5], plus an item on grip strength (`Do you have any difficulty gripping with your hands (e.g. opening a jam jar)?).

Personal data and linked study data will be stored on University computers, encrypted and password protected, with regular back up, in a searchable format and may be used to screen potential participants for eligibility for nested research studies, and to enable contact for invitation to participation.

### Quantitative variables

The data collected are summarised in Table 1. Joint pain severity will be reported using the numerical rating scale from 0 to 10. Pain quality will be classified under sensory, affective and evaluative domains using word descriptors from the McGill Pain Questionnaire, also permitting calculation of domain-specific pain severity [35]. Central aspects of pain will be calculated from CAP-Knee responses as a self-report index of central sensitisation [36]. Self-reported current medication use will be classified based on Bedson et al., 2013, to include categories of non-steroidal anti-inflammatory drugs (NSAIDs), paracetamol, opiates or medications recommended by NICE for neuropathic pain [37, 38].

Frailty will be assessed using the FRAIL scale [39], combining the 3 items addressing Fatigue, Resistance (climbing steps), Ambulation (walking), together with weight loss and morbidity counts. People fulfilling 1 or 2 criteria may be classified as “pre-frail” and 3 or more as frail. Secondary analyses of frailty will be based on 2 other self-report systems. The first uses criteria proposed by Fried et al. [40], (weight loss, fatigue, ambulation, weakness and inactivity) where individuals are classified as “pre-frail” (1–2 criteria) or frail (3 or more). The other uses items corresponding to those included within the Frail Non Disabled (FiND) questionnaire [41] where people may be classified as disabled (problems with ambulation), frail (no ambulation problems but exhibits weight loss, fatigue or inactivity) or robust (none).

Key domains linked to joint pain, frailty and disability will include multimorbidity and fracture risk. Multimorbidity will be assessed using morbidity counts informed by the derivations of the Charlson Comorbidity Index [42] and Rheumatic Disease Comorbidity Index (RDCI) [43]. Morbidities will be identified using a structured check list supplemented by participants’ free text self-reports identifying specific MSK and non-MSK morbidities. Fracture risk will be assessed based on the chart-based FRAX^R^ fracture risk assessment tool [44–46]. In people without bone mineral density measurements, the BMI and other risk factors are cross-referenced. We will determine FRAX risk scores using self-reported osteoporosis, use of anti-osteoporotic medications, and exposure to systemic glucocorticoids, current smoking, intake of alcohol and comorbidities indicative of secondary osteoporosis. Family fracture history will not be available.

### Statistical methods

The IMH&W questionnaire is constructed from items and validated questionnaires used in previous research studies, and analyses will be undertaken at item and questionnaire levels. Descriptive data will be presented as mean (SD) or median (IQR). Statistical analyses may be complemented by qualitative analysis of descriptors/vocabulary sets and narremes [47] used in free-text responses.

*Primary objective: to measure and characterise the development and progression of pain, frailty and disability and their associations in a cohort of adults with MSK problems.*

Prevalence will be reported of clinical characteristics within the study population. Changes in pain, frailty and disability will be measured over time, using both dichotomous and continuous variables. For example, rates of transition between robust, pre-frail and frail categories will be determined [6, 40]. Similarly transitions between MSK pain-free and painful categories will be determined, based on responses the question `over the past 4 weeks, have you had pain or aching in any of your joints?’. Changes in severity or quality of pain will be evaluated using continuous questionnaire measures, Rasch transformed where appropriate to the calculation of change data. Change scores will be adjusted for cognate baseline scores. Loss to follow-up rate between successive questionnaire phases will be investigated for possible associations with factors such as age, gender, recruitment pathway, frailty and symptom severity.

The sampling strategy is one of cluster sampling and as such the errors terms will be adjusted for clustering or/and multi-level models will be run where required. Multiple Imputation will only be used if the conditions support it, such as the pattern and predictors of missing data and the percentage of missing data being small [48]. We will use a pattern match to account for non-random missing data [48] Kaplan-Meier analysis will generate survival curves for binary outcomes, and time-to-event outcomes for multiple follow-up data will use log-rank tests. The Cox proportional hazards model will be used to calculate hazard ratios (HR), adjusted for confounding factors.

IMH&W questionnaire internal validity and structure will be determined. Rasch analysis of CAP-Knee questionnaire items will be conducted in order to determine validity within the IMH&W population. Response dependencies between items will be identified from residual correlation matrices. Summary fit residuals (mean ± SD) for items and persons, and Chi-square testing for item–trait interactions, will be used to evaluate overall fit to the Rasch model. Item subsets will be checked for disordered response thresholds and DIF for sex, age and relevant disease characteristics.

*Secondary objective 1. To identify discrete participant subgroups and their associations based on baseline characteristics and on pain, frailty and disability trajectories.*

Allocation to participant subgroups will be based on questionnaire responses and recruitment pathways. Socioeconomic status will be derived from postcode data using GeoConvert [49] to determine the English Index of Multiple Deprivation [50]. Participant subgroups may also be determined empirically using Latent Class Analysis or Group-Based Trajectory Analysis. Variation between subgroups and across time will be evaluated using parametric or non-parametric statistical methods as appropriate. Linear multivariable regression models will be used to identify baseline predictors of pain, frailty and disability outcomes and trajectories. Potential predictors may include demographic and clinical characteristics, recruitment pathway and wave. Potential moderator roles, for example of central pain mechanisms, may be assessed. Risk factors associated with subgroup allocation will be examined. Odds ratio (OR) and 95% confidence interval (CI) will be given to present associations. All models will be adjusted for potential confounding factors and checked for interactions and collinearity as appropriate. Logistic regression models will be used to adjust for confounding factors such as age, gender, and body mass index.

*Secondary objective 2. To facilitate nested qualitative, observational and interventional research studies by providing information required for study design, and by permitting the identification and recruitment of eligible research study participants.*

Recruitment rate, efficiency, retention and costs will be determined based on screened populations, dispatched invites and IMH&W study participation numbers. Recruitment efficiency and retention will be compared between recruitment pathways and between demographic and clinical subgroups. Recruitment from IMH&W to nested studies also will be evaluated, and compared between IMH&W participant subgroups. Nested research projects may include further surveys, qualitative research, clinical trials or mechanistic studies. Weighting, or other methodologies might be employed in analyses to address differential recruitment rates and longitudinal attrition.

*Secondary objective 3. To characterise the longitudinal context and recruitment biases of nested research studies in order to better inform generalisation from research findings, to provide long-term observational follow up data, and to provide a source cohort for cohort randomized controlled trials (RCTs).*

IMH&W participants may be recruited to future interventional RCTs, dependent on specific ethical and governance approvals, and specific consent for each trial. IMH&W may provide source cohort data, by matching IMH&W participants to nested trial participants. Linkage of nested trial participant identifiers to IMH&W identifiers may permit follow up beyond the duration of the nested study. Follow up data collection within IMH&W and nested studies will converge, both in timing and data content. Anonymised data from IMH&W may be made available to nested studies according to their protocol, ethical and governance approvals. Differences between study populations (those invited to participate and responders) will be tested.

Statistical significance will be inferred when the *P* value is less than 0.05, or when 95% CI does not include unity.

### Sample size

A recruitment target of 10,000 people has been set, based on feasibility and previous data from similar surveys within the East Midlands. Our recent community-based cohort (Knee Pain in the Community, KPIC) also had an objective to recruit to nested research studies [51]. Nine thousand four hundred eighty-five participants returned baseline questionnaires, of whom 6714 agreed to future contact. Baseline questionnaires permitted recruitment of 219 participants with knee pain onset within 3 years, providing sufficient power for a study of deep phenotyping [51]. From this experience, approximately 80 GP practices located throughout the East Midlands region may be required for recruitment to IMH&W.

Selection of participants for future studies, RCTs and cohort RCTs will depend on specific characteristics defined by questionnaire responses. Numbers required for each future study will depend on the design of that study, and recruitment efficiency. Studies of rare or `seldom heard groups’ participant subgroups, and studies that apply more rigorous eligibility and exclusion criteria, will require a larger total database from which to identify eligible participants for intervention.

## Discussion

IMH&W is a prospective cohort study aiming to elucidate associations and progression of pain, frailty and disability. It will enable identification of people at risk of poor MSK health and wellbeing who might be suitable for specific interventions, and facilitate generalisation and comparison of research outcomes between target populations. Identified modifiable risk factors might indicate novel treatment targets or management strategies that can reduce the burden of MSK conditions. IMH&W will elucidate recruitment bias in nested studies, and aid development of targeted recruitment strategies that minimise such bias. Quantifying changes in measured outcomes over time will support power calculations for studies of interventions aiming to improve those outcomes. Identifying participant subgroups will facilitate study design for research on targeted interventions.

Previous cohorts have identified predictors of MSK pain, frailty or disability [51–53]. Some factors, such as age or activity, might predict all of these outcomes, whereas others, like early menopause, might predict fracture risk but not pain nor disability. Few cohorts enable investigation of all 3 of these key MSK outcomes, and the apparent differences between risk factors or outcomes might be attributed to different study designs or populations. Disease specific cohorts (e.g. inception cohorts of people with rheumatoid arthritis [54]), do not permit comparisons between diagnostic groups, whereas even very large population cohorts may have insufficient power to permit robust conclusions on important participant subgroups. IMH&W attempts to address some of these limitations by standardised measurement of pain, disability and frailty in all participants, and recruiting from diverse populations, targeted to MSK problems but not restricted to specific disease groups.

Currently there is a perceptible social gradient in the distribution of good health, with those living in poorer areas and at the lower end of the social gradient on average experiencing shorter life spans, inferior health and more years of disability [55]. Furthermore, the heterogeneity of outcomes may also be exacerbated with age and in different ethnic groups [56, 57] Recruitment of a large cohort through diverse pathways across a broad geographic area will facilitate evaluation of effects of inequalities on the progression of MSK pain, disability and frailty. Analysis of medication use and comorbidities may help identify some of the underlying causes, and increased understanding should help design strategies to address pain, disability and frailty in different social contexts, and to reduce health inequalities.

Recruitment to nested studies from the IMH&W population should increase recruitment efficiency compared to less targeted recruitment. Use of a prepopulated participant contact list will permit timely recruitment that is not dependent on health care attendances. Furthermore, recruitment could be based on characteristics like pain, disability and frailty, which are not dependent on diagnostic classification by health care providers. Targeted recruitment should minimise burden to non-eligible people. A better understanding of the size of eligible populations will facilitate realistic study design. Information from the IMH&W cohort should reassure funders, ethics committees and participants that the scientific outcomes of proposed research are realistic and achievable.

IMH&W will benefit from a flexible study design. Over the lifespan of a cohort, new knowledge, and demographic and societal shifts can necessitate study protocol refinement. For example, extended or enriched recruitment of specific participant groups was required to quantify risks in newer groups of people exposed to biologic agents in the British Society for Rheumatology Biologics Register [58]. Successive waves of data collection may address new domains of recent interest [59]. This exploratory nature of cohort studies can limit interpretation of their findings, in that even very large cohorts might be underpowered for multiple post hoc analyses, the total number of which is unknown at cohort inception. This published protocol aims to give not only an understanding of our cohort design, but also permit open evaluation of which of our analyses are primary or exploratory.

### Limitations

IMH&W will be subject to several limitations. This will not be a representative cohort, but purposive recruitment will enrich the cohort with people with MSK problems, possibly with high representation of elderly and unwell people. As such, although incidence and prevalence of specific problems may be measured within the IMH&W cohort, including within subpopulations recruited through discrete pathways, extrapolation of these data to the source populations will be limited. Cohort studies are themselves subject to recruitment bias. Long questionnaires and face:face assessments can provide rich data from participants, but their burden might reduce recruitment rates. The IMH&W questionnaire is designed to be easy and quick to complete in order to maximise responses and minimise participant burden, but, as a result, unmeasured variables may confound our findings. People might be more willing to consider participation in research when they are receiving clinical care, either out of gratitude (`to give something back’) or in the hope of personal clinical benefit. Community cohorts might have lower recruitment rates than do hospital-based cohorts. The multiple recruitment sources used in IMH&W will enable us to investigate these possible differences in recruitment. The IMH&W questionnaire has been compiled from previously validated items and questionnaires, but self-reported data are liable to recall biases and inaccuracies, and some characteristics may only be approximately measured.

## Conclusions

To conclude, IMH&W is a questionnaire-based cohort study which aims to elucidate associations and progression of pain, frailty and disability, to identify risk factors for poor outcomes and facilitate the execution and interpretation of research in order to improve outcome in specific populations where there is a particular need.

## Supplementary information

**Additional file 1.**

**Additional file 2.**

## Data Availability

Not applicable. The manuscript does not report data. The datasets subsequently generated and/or analysed during the current study may be made publicly available following conclusion of ongoing research. Requests for data may be made at any time to the corresponding author.

## Abbreviations

MSK: Musculoskeletal
IMH&W: Investigating Musculoskeletal Health and Wellbeing
PIS: Participant Information Sheet
RCT: Randomised Controlled Trial
eFI: e-Frailty Index
RDCI: Rheumatic Disease Comorbidity Index
GCP: Good Clinical Practice
NSAID: Non-steroidal anti-inflammatory drug
FIND: Frail Non Disabled scale
