# Supplementary material for: Investigating musculoskeletal health and wellbeing; a cohort study protocol
Source: BMC Musculoskelet Disord. 2020 Mar 21;21:182. doi: 10.1186/s12891-020-03195-4 (PMC7085148; doi:10.1186/s12891-020-03195-4)
Supplement: Supplementary file 1 — Additional file 1. [file 12891_2020_3195_MOESM1_ESM.docx]

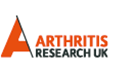

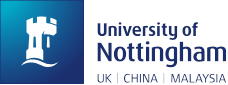

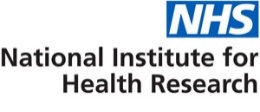
**Nottingham**

**Biomedical Research Centre**

**Investigating Musculoskeletal Health and Wellbeing**

We are interested in learning why some people experience joint pains, weakness or falls, whereas others remain fit and well into later life. We are investigating ways of preventing or treating these health issues.

We would be very grateful if you would take the time to complete this questionnaire, **regardless of whether or not you have suffered from these problems**. Replies from all people are important and useful for this research, even if you have not answered all of the questions.

Please return this questionnaire, in the pre-paid envelope **(no stamp required)** to the University of Nottingham as soon as possible.

**Your answers are strictly confidential**

If you have any questions or require any advice on completing the questionnaire please telephone our Research Coordinator and Study Contact Bonnie Millar on 0115-8231676, [msk-recruitment@nottingham.ac.uk](mailto:msk-recruitment@nottingham.ac.uk).

**Thank you for your assistance with this important area of research.**

**Chief Investigator –** Professor David Walsh

**Approved by:** London Central Research Ethics Committee

**Funded by: National Institute for Health Research and Arthritis Research UK**

| *Office use only* | |  |  |  |  |  |  |  |  |  |
| --- | --- | --- | --- | --- | --- | --- | --- | --- | --- | --- |
| Person ID |  |  |  |  |  |  | Surgery/PIC ID |  |  |  |

**Section A: Information about you and your treatments**

What is your **date of birth**?

|  |  | Day |  |  |  | Month |  |  |  |  |  | Year |
| --- | --- | --- | --- | --- | --- | --- | --- | --- | --- | --- | --- | --- |

What is your **sex**?

|  | Male |  | Female |  | Prefer not to say |
| --- | --- | --- | --- | --- | --- |

Choose one option that best describes your **ethnic group** or **background?**

|  | White |  | Asian |  | Black |
| --- | --- | --- | --- | --- | --- |

| Any other ethnic group (please describe) | | | | |  |  |  |
| --- | --- | --- | --- | --- | --- | --- | --- |
|  |  |  |  |  |  |  |  |
|  |  |  |  |  |  |  |  |

What is your **height**?

|  | Feet |  |  |  |  | inches | **OR** |  |  |  | centimetres |
| --- | --- | --- | --- | --- | --- | --- | --- | --- | --- | --- | --- |

How much do you **weigh** with your clothes on but without shoes?

|  |  | stones |  |  | pounds | **OR** |  |  |  | kilograms |
| --- | --- | --- | --- | --- | --- | --- | --- | --- | --- | --- |

**One year ago** how much did you **weigh** without shoes but with your clothes on?

|  |  | stones |  |  | pounds | **OR** |  |  |  | kilograms |
| --- | --- | --- | --- | --- | --- | --- | --- | --- | --- | --- |

What is your **smoking** status?

|  | Smoker |  | Ex-smoker **OR** |  | Never smoked |
| --- | --- | --- | --- | --- | --- |

Do you drink usually 3 units or more of **alcohol** per day?

*3 units might, for example, be 2 pints of lager, 2 glasses of wine or 3 single shots of spirit.*

|  | Yes |  | No |
| --- | --- | --- | --- |

**Has a doctor told you that you have any of these medical conditions or problems?**

**If yes, please place a tick in the boxes provided.**

|  | Angina |  | Heart attack | |  | | |  |  |
| --- | --- | --- | --- | --- | --- | --- | --- | --- | --- |
|  | Arthritis |  | Heart failure | |  | | |  |  |
|  | Asthma |  | Hypertension | |  | | |  |  |
|  | Back or spine problems |  | Kidney disease | |  | | |  |  |
|  | Cancer (not minor skin cancers) |  | Lung disease | |  | | |  |  |
|  | Dementia |  | Osteoarthritis | |  | | |  |  |
|  | Diabetes mellitus |  | Osteoporosis | |  | | |  |  |
|  | Fibromyalgia |  | Rheumatoid arthritis | |  | | |  |  |
|  | Gout |  | Stroke | |  | | |  |  |
|  |  |  |  |  |  |  |  | |  |

| Others (please specify any conditions not listed above) | | |  | | |  |  | | |
| --- | --- | --- | --- | --- | --- | --- | --- | --- | --- |
|  |  |  | |  |  | | |  |  |
|  |  |  | |  |  | | |  |  |
|  |  |  | |  |  | | |  |  |
|  |  |  | |  |  | | |  |  |
|  |  |  | |  |  | | |  |  |

**Medicines including pain killers**

**Please write down the names of any medications, including any pain killers, that you use. They can be prescriptions or bought over the counter.**

Names of medications

|  |  | Never | Sometimes | Often | Always |
| --- | --- | --- | --- | --- | --- |
| 1. | Cold or heat (e.g. bath water) on my knee was painful |  |  |  |  |
| 2. | I generally felt tired |  |  |  |  |
| 3. | Knee pain stopped me concentrating on what I was doing |  |  |  |  |
| 4. | I kept thinking about how much my knee hurts |  |  |  |  |
| 5. | In general, I got sudden feelings of panic |  |  |  |  |
| 6. | Knee pain affected my sleep |  |  |  |  |
| 7. | I generally still enjoyed the things I used to enjoy |  |  |  |  |
| 8. | This final question is about pain you may have had in any part of your body. Please shade in the diagram below, to indicate where you have suffered any pain for most days in the last ***4 WEEKS***. By pain we also mean aching and/or discomfort. Please do not include pain due to feverish illness such as flu. | | | | |

**Section B: CAP-Knee (Central Aspects of Pain in Knee) Scale**

**Please select the response that best describes how you have felt over the PAST *WEEK*. Please tick one box only per statement and try not to leave any statements blank.**

R

L

R

L

**Section C: Joint Aches and Pains**

1. Over the **past 4 weeks**, have you had **pain** or **aching** in **any of your joints**?

|  | Yes |  |  | No |  | If you answer ‘No’, please move to section D. |
| --- | --- | --- | --- | --- | --- | --- |

**Most bothersome joint**

2. Over the **past 4 weeks**, where was your most bothersome joint pain or aching feeling? (Pick **one**)

|  | Jaw |  |  | Wrist |  |  | Neck |
| --- | --- | --- | --- | --- | --- | --- | --- |
|  | Back or spine |  |  | Knee |  |  | Ankle |
|  | Shoulder |  |  | Hand or finger |  |  | Foot or toe |
|  | Elbow |  |  | Hip |  |  |  |

3. Over the **past 4 weeks**, how intense was your **average pain** or the **average aching feeling in your most bothersome joint,** where 0 is ‘no pain’ and 10 is ‘pain as bad as could be’?

No pain Pain as bad as could be

| 0 | 1 | 2 | 3 | 4 | 5 | 6 | 7 | 8 | 9 | 10 |
| --- | --- | --- | --- | --- | --- | --- | --- | --- | --- | --- |

4. Was your **most bothersome join**t painful for **most days** of the last **4 weeks**?

|  | Yes |  |  | No |  |  |
| --- | --- | --- | --- | --- | --- | --- |

5. What does your pain **feel** like? Tick those words that best describe it. Leave out any category that is not suitable. Use only **one** word in each category.

| 1 | 2 | 3 | 4 | 5 | 6 | 7 |
| --- | --- | --- | --- | --- | --- | --- |
| Flickering | Jumping | Pricking | Sharp | Pinching | Tugging | Hot |
| Quivering | Flashing | Boring | Cutting | Pressing | Pulling | Burning |
| Pulsing | Shooting | Drilling | Lacerating | Gnawing | Wrenching | Scalding |
| Throbbing |  | Stabbing |  | Cramping |  | Searing |
| Beating |  | Lancinating |  | Crushing |  |  |
| Pounding |  |  |  |  |  |  |
|  |  |  |  |  |  |  |
| 8 | 9 | 10 | 11 | 12 | 13 | 14 |
| Tingling | Dull | Tender | Tiring | Sickening | Fearful | Punishing |
| Itchy | Sore | Taut | Exhausting | Suffocating | Frightful | Grueling |
| Smarting | Hurting | Rasping |  |  | Terrifying | Cruel |
| Stinging | Aching | Splitting |  |  |  | Vicious |
|  | Heavy |  |  |  |  | Killing |
|  |  |  |  |  |  |  |
| 15 | 16 | 17 | 18 | 19 | 20 |  |
| Wretched | Annoying | Spreading | Tight | Cool | Nagging |  |
| Blinding | Troublesome | Radiating | Numb | Cold | Nauseating |  |
|  | Miserable | Penetrating | Drawing | Freezing | Agonizing |  |
|  | Intense | Piercing | Squeezing |  | Dreadful |  |
|  | Unbearable |  | Tearing |  | Torturing |  |

6. Any other comments?

**Section D: Your activities and general health**

1. Which is your level of physical activity?

|  | Regular physical activity  (at least 2-4 hours per week) |  |  | None or mainly sedentary |  |  |
| --- | --- | --- | --- | --- | --- | --- |

2. By yourself and not using aids, do you have any difficulty walking several hundred yards?

|  | Yes |  |  | No |  |  |
| --- | --- | --- | --- | --- | --- | --- |

3. By yourself and not using aids, do you have any difficulty walking up 10 steps without resting?

|  | Yes |  |  | No |  |  |
| --- | --- | --- | --- | --- | --- | --- |

4. Do you have any difficulty gripping with your hands (e.g. opening a jam jar)?

|  | Yes |  |  | No |  |  | Some |
| --- | --- | --- | --- | --- | --- | --- | --- |

5. How much of the time during the past 4 weeks did you feel tired?

|  | All of the time |  | Most of the time |  | Some of the time |  | A little of the time |  | None of the time |
| --- | --- | --- | --- | --- | --- | --- | --- | --- | --- |

Thank you very much for taking the time to complete this questionnaire.
